# The Changing Concept in the History of Schizophrenia

**DOI:** 10.3390/brainsci16050447

**Published:** 2026-04-23

**Authors:** Eugenio Cavalli, Giuseppe Rosario Pietro Nicoletti, Ferdinando Nicoletti

**Affiliations:** Department of Biomedical and Biotechnological Sciences, University of Catania, 95123 Catania, Italy; eugeniocavalli9@hotmail.it (E.C.); giunicol01@gmail.com (G.R.P.N.)

**Keywords:** schizophrenia, history of medicine, history of psychiatry, diagnostic evolution, nosography, psychiatric classification, biomarkers, heterogeneity

## Abstract

**Highlights:**

**What are the main findings?**

- The concept of schizophrenia has evolved through successive historical paradigms, from descriptive psychopathology to operational, biological, and computational models, each contributing to increased diagnostic reliability while also increasing clinical heterogeneity.
- Despite significant advances in neurobiology, genetics, and digital psychiatry, no single biomarker has demonstrated sufficient specificity, supporting the view of schizophrenia as a biologically heterogeneous and historically evolving clinical construct.

**What are the implications of the main findings?**

- Future research should prioritize dimensional, stratification-based, and multimodal approaches integrating clinical, biological, and digital data beyond categorical diagnostic frameworks.
- A historically informed perspective is essential to properly interpret current findings and to develop flexible diagnostic frameworks capable of balancing biological complexity with clinical utility.

**Abstract:**

**Background/Objectives**: Schizophrenia is one of the most extensively studied yet conceptually unstable disorders in the history of medicine and brain sciences. Since its formalization at the turn of the twentieth century, the disorder has been repeatedly redefined, reflecting changes in clinical observation, diagnostic philosophy, and neuroscientific models of brain function. The objective of this review is to critically examine the historical evolution of schizophrenia as a medical construct and to analyze how shifts in diagnostic systems have shaped the search for biological and molecular biomarkers. **Methods**: A narrative-historical review of the literature was conducted, integrating classical psychiatric texts, diagnostic manuals, and contemporary neuroscientific studies. Key milestones in the conceptualization of schizophrenia were analyzed alongside the development of biological hypotheses, including neurochemical, electrophysiological, neuroimaging, genetic, immunological, omics-based, and digital approaches. Emphasis was placed on identifying conceptual continuities, ruptures, and methodological limitations across historical periods. **Results**: The analysis reveals that the evolution of schizophrenia has been characterized by increasing diagnostic standardization accompanied by growing biological heterogeneity. While successive biological models have provided valuable insights into specific aspects of the disorder, none have yielded single, robust diagnostic biomarkers. Instead, findings consistently reflect partial overlaps between clinical phenotypes and biological signals, strongly influenced by historically derived diagnostic categories. **Conclusions**: The persistent absence of definitive diagnostic biomarkers for schizophrenia reflects not only technical limitations but also the historical construction of the disorder as a heterogeneous clinical category. Understanding this historical context is essential for interpreting current findings in brain sciences. Future research is likely to benefit from stratification-based, dimensional, and integrative frameworks that move beyond categorical diagnosis while preserving clinical relevance.

## 1. Introduction

Schizophrenia occupies a singular position in the history of medicine, representing one of the most enduring and debated diagnostic constructs in the study of mental and brain disorders. Since its formalization at the end of the nineteenth century, schizophrenia has been repeatedly redefined, reflecting broader transformations in medical epistemology, clinical practice, and conceptions of the relationship between mind and brain. Its history offers a privileged lens through which to examine how medical knowledge is constructed, stabilized, and periodically revised in response to changing theoretical frameworks and technological advances.

Long before the emergence of modern psychiatry, clinical phenomena later subsumed under the diagnosis of schizophrenia were interpreted through moral, religious, or philosophical paradigms. The gradual shift toward medical observation, institutional care, and longitudinal clinical description marked a crucial turning point in the nineteenth century, when mental disorders began to be conceptualized as distinct disease entities. This process culminated in the development of descriptive psychopathology and nosographic systems, most notably with the work of Emil Kraepelin, who framed dementia praecox as a disorder defined by course and prognosis rather than isolated symptoms. Subsequent reformulations, particularly those introduced by Eugen Bleuler, further transformed schizophrenia into a heterogeneous clinical construct centered on core psychopathological features.

Throughout the twentieth century, efforts to standardize psychiatric diagnosis led to the progressive codification of schizophrenia within international classification systems, including the Diagnostic and Statistical Manual of Mental Disorders (DSM) and the International Classification of Diseases (ICD). These systems prioritized diagnostic reliability and operational criteria, profoundly influencing clinical practice and research design. At the same time, they contributed to an expansion of diagnostic boundaries and increasing recognition of heterogeneity, raising persistent questions about the validity of schizophrenia as a unified disease entity.

Parallel to these nosographic developments, successive biological hypotheses—ranging from neurochemical and electrophysiological models to neuroimaging, genetic, immunological, and omics-based approaches have sought to anchor schizophrenia to identifiable biological substrates. Despite substantial advances in brain sciences, no single biomarker has demonstrated sufficient specificity or robustness to serve as a definitive diagnostic tool. This recurring failure suggests that the problem is not solely methodological but is deeply intertwined with the historical evolution of the diagnostic construct itself.

The aim of this review is to provide a critical, narrative-historical analysis of schizophrenia as a medical concept, tracing its evolution from pre-nosographic interpretations to contemporary diagnostic frameworks and biomarker research. This narrative review was conducted through a structured selection of historical psychiatric texts, diagnostic manuals, and contemporary neuroscientific literature, prioritizing influential conceptual developments and representative biological research across different historical periods. Although not designed as a systematic review, this approach aimed to provide a comprehensive and historically grounded synthesis of the evolving concept of schizophrenia. By integrating historical, clinical, and neuroscientific perspectives, this review seeks to elucidate how the historical construction of schizophrenia has shaped current research paradigms, to identify enduring conceptual and methodological gaps, and to outline historically informed directions for future research in brain sciences.

## 2. Pre-Nosographic Observations

Before the emergence of modern psychiatric nosography, clinical phenomena that would later be subsumed under the diagnosis of schizophrenia were interpreted within explanatory frameworks fundamentally different from contemporary medical models. In classical antiquity, disturbances of thought, perception, and behavior were addressed through a combination of philosophical reasoning and early medical theory. Hippocratic medicine marked a decisive departure from supernatural explanations by attributing mental disturbances to bodily imbalances, particularly within the humoral framework, thereby embedding disorders of the mind within a general somatic theory of disease [1]. Parallel philosophical traditions, notably those of Plato and Aristotle, conceptualized madness as a disruption of rational faculties, oscillating between pathological states and forms of divinely inspired experience [2,3].

Galenic physiology further systematized humoral explanations and dominated medical thought well into the Middle Ages, reinforcing a holistic conception of mental disturbance grounded in bodily processes [4]. At the same time, Christian theology reintroduced moral and spiritual interpretations, frequently framing madness in terms of sin, possession, or divine trial. Despite this moralization, medieval medical practice preserved elements of empirical observation, particularly within monastic infirmaries and early hospitals, where care of the mentally disturbed was integrated into broader medical and charitable activities [5].

A gradual epistemic shift occurred between the late medieval period and the Enlightenment, as naturalistic and observational approaches gained prominence. Renaissance humanism and advances in anatomy renewed interest in the brain as the anatomical substrate of cognition and behavior, although clear distinctions between neurological and mental disorders remained poorly defined [6]. By the seventeenth and eighteenth centuries, physicians increasingly emphasized systematic observation, clinical description, and attempts at classification. Terms such as *melancholia*, *mania*, and *insanity* were employed descriptively, yet their meanings remained fluid and context-dependent, reflecting the absence of standardized diagnostic criteria [7].

The emergence of asylums in early modern Europe represented a critical institutional transformation in the medical understanding of mental disorders. Initially conceived as spaces of confinement rather than therapeutic intervention, asylums nonetheless enabled prolonged and repeated observation of patients over time. This longitudinal perspective constituted a methodological innovation, allowing physicians to identify recurring symptom patterns and disease trajectories [8]. Reformers such as Philippe Pinel and Jean-Étienne Esquirol emphasized careful clinical observation, symptom grouping, and differentiation of mental conditions based on observable features, marking a decisive move toward medicalization [9].

Crucially, pre-nosographic approaches did not conceptualize mental disorders as discrete, stable disease entities. Instead, they reflected a continuum of mental phenomena interpreted through prevailing cultural, philosophical, and medical lenses. While this flexibility limited diagnostic precision, it avoided rigid categorical boundaries that would later dominate psychiatric classification [10]. Beyond European traditions, early conceptualizations of mental disturbances also emerged in non-Western medical systems [11]. Traditional Chinese Medicine described severe disturbances of thought and behavior within frameworks involving imbalances of qi, yin-yang, and organ systems, particularly the heart and liver, which were considered central to mental functioning [12]. Similarly, medieval Islamic medicine provided important contributions to the biological understanding of psychiatric disorders. Physicians such as Al-Razi (Rhazes) described mental disturbances using medical and observational approaches and helped establish some of the first hospitals with dedicated psychiatric wards in cities such as Baghdad and Cairo [13]. These early cross-cultural perspectives contributed to the gradual medicalization of mental disorders and anticipated later developments in psychiatric nosography. The enduring legacy of this period lies not in specific diagnostic labels, but in the establishment of key methodological principles systematic observation, longitudinal follow-up, and descriptive analysis that directly informed the development of descriptive psychopathology and the subsequent emergence of modern nosographic systems in the nineteenth century [14].

## 3. Nineteenth Century and the Birth of a Syndrome

This The nineteenth century marked a decisive turning point in the medical conceptualization of severe mental disorders, with the emergence of descriptive psychopathology and the systematic classification of psychiatric conditions. Advances in clinical medicine, pathological anatomy, and institutional psychiatry fostered a new emphasis on careful observation, longitudinal follow-up, and the identification of recurring patterns of symptoms and disease course. Within this context, mental disorders began to be framed as distinct clinical entities rather than as loosely defined states of madness [10,15].

Emil Kraepelin played a central role in this transformation by introducing a nosographic system grounded not primarily in symptom content, but in the temporal evolution of illness. Through meticulous clinical observation of asylum populations, Kraepelin distinguished *dementia praecox* from manic–depressive illness, arguing that the former was characterized by an early onset and a progressive deterioration of cognitive and functional capacities [16]. Course and prognosis thus became defining diagnostic criteria, marking a departure from purely cross-sectional descriptions of mental symptoms.

Kraepelin’s approach reflected broader methodological shifts in nineteenth-century medicine, in which diseases were increasingly defined by their natural history rather than by isolated manifestations. His conceptualization of *dementia praecox* integrated heterogeneous clinical presentations such as catatonia, hebephrenia, and paranoid psychosis—under a single diagnostic umbrella, unified by a presumed common trajectory [17]. While this synthesis provided a powerful organizational framework, it also introduced an inherent tension between clinical heterogeneity and diagnostic unity. The influence of Kraepelin’s nosography extended far beyond his own work, shaping subsequent psychiatric classification systems and research agendas throughout the twentieth century. At the same time, the emphasis on prognosis as a defining feature would later be challenged, as accumulating clinical evidence revealed substantial variability in outcomes [18]. Nonetheless, Kraepelin’s formulation of *dementia praecox* represents a foundational moment in the history of schizophrenia, establishing the conceptual and methodological basis upon which later revisions and critiques would build [19].

## 4. Bleuler and the Concept of Schizophrenia

The beginning of the twentieth century witnessed a major conceptual revision of Kraepelin’s formulation of dementia praecox, most notably through the work of Eugen Bleuler. While acknowledging Kraepelin’s clinical insights, Bleuler challenged the centrality of inevitable deterioration and early onset as defining features of the disorder. Based on extensive clinical observation, he argued that the course of illness was far more variable than previously assumed and that many patients did not progress toward irreversible cognitive decline [17]. This critique marked a decisive shift away from prognosis as the primary organizing principle of diagnosis.

**Figure 1:**
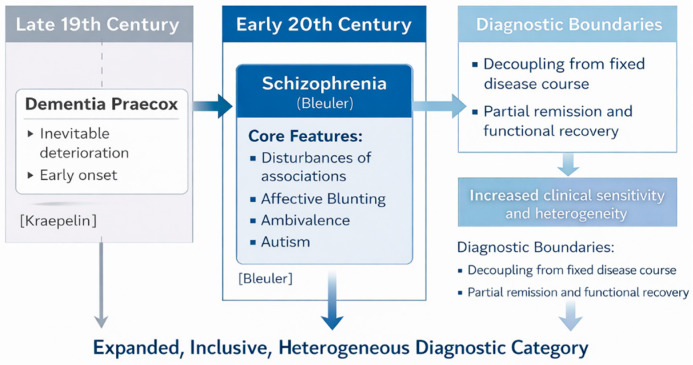
**Conceptual shift from Kraepelin’s dementia praecox to Bleuler’s concept of schizophrenia.** The figure illustrates the transition from Kraepelin’s late nineteenth-century formulation of dementia praecox, defined by early onset and inevitable deterioration, to Bleuler’s early twentieth-century reconceptualization of schizophrenia. Bleuler emphasized fundamental disturbances of psychic functions (the “four As”: associations, affect, ambivalence, and autism) and decoupled the diagnosis from a fixed disease course, allowing for partial remission and functional recovery. This shift resulted in an expanded and more inclusive, but also more heterogeneous, diagnostic category, with lasting implications for psychiatric classification.

In 1911, Bleuler introduced the term *schizophrenia* to replace *dementia praecox*, emphasizing the fragmentation (*Spaltung*) of psychic functions rather than dementia as the core pathological process [20]. This terminological change reflected a deeper theoretical reorientation, in which schizophrenia was conceptualized as a disorder of fundamental psychological processes rather than a unitary degenerative disease. Bleuler identified a set of core features later known as the “four As”: disturbances of associations, affective blunting, ambivalence, and autism which he considered essential to the diagnosis, while regarding hallucinations and delusions as secondary or accessory symptoms [21]. This terminological and conceptual shift marked a profound redefinition of the disorder, moving the focus from an inevitably degenerative disease course to fundamental disturbances of psychic functions and greater clinical variability (Figure 1).

Bleuler’s formulation significantly broadened the diagnostic boundaries of schizophrenia. By decoupling the disorder from a fixed disease course and allowing for partial remission and functional recovery, his model increased clinical sensitivity but also introduced greater heterogeneity [22]. The emphasis on underlying psychological mechanisms, influenced in part by emerging psychodynamic theories, further distanced schizophrenia from strictly somatic or neuropathological explanations. As a result, schizophrenia became a more inclusive and flexible diagnostic category, encompassing a wide range of clinical presentations.

This conceptual expansion had lasting consequences for psychiatric classification. While Bleuler’s approach enriched clinical understanding and reduced the pessimism associated with Kraepelin’s model, it also weakened diagnostic specificity and blurred the boundaries between schizophrenia and other psychotic or personality-related conditions [23]. From a contemporary neurobiological perspective, Bleuler’s conceptual expansion may also be viewed as a critical turning point in the biological fragmentation of schizophrenia. By prioritizing diagnostic sensitivity over specificity, the Bleulerian model broadened clinical boundaries and incorporated heterogeneous phenotypes under a single diagnostic label [24,25]. While this inclusivity improved clinical recognition, it also introduced substantial biological variability. Such heterogeneity poses a major challenge for biomarker discovery, as biologically distinct subgroups are aggregated within a single diagnostic category [26,27]. Consequently, the search for single biological markers or unified pathophysiological mechanisms may be intrinsically limited by the historical expansion of diagnostic criteria. This trade-off between clinical inclusivity and biological precision represents a central tension that continues to shape contemporary schizophrenia research. The tension between inclusivity and precision introduced by Bleuler would persist throughout the twentieth century, shaping subsequent debates on diagnosis, classification, and the search for biological correlates.

In retrospect, Bleuler’s reconceptualization represents a pivotal moment in the history of schizophrenia, transforming it from a narrowly defined disease entity into a broad psychopathological construct. This shift laid the groundwork for later diagnostic systems and simultaneously contributed to the enduring problem of heterogeneity that continues to challenge contemporary brain sciences [28].

## 5. Schneider and First-Rank Symptoms

In the mid-twentieth century, the conceptualization of schizophrenia underwent a further transformation through the work of Kurt Schneider, who sought to address the growing diagnostic ambiguity introduced by Bleuler’s broadened definition. While Bleuler’s emphasis on fundamental psychological disturbances enriched clinical understanding, it also increased heterogeneity and reduced diagnostic specificity. Schneider’s contribution can be understood as an attempt to restore diagnostic clarity by identifying symptoms that were highly characteristic of schizophrenia and could be reliably recognized across clinicians and settings [10,29].

Schneider introduced the concept of *first-rank symptoms* (FRS), a group of psychotic phenomena he considered particularly indicative of schizophrenia. These included experiences such as auditory hallucinations commenting or conversing, thought insertion, thought withdrawal, thought broadcasting, and delusional perception. Unlike Bleuler’s core features, which emphasized underlying psychological processes, Schneider’s symptoms were defined phenomenologically and prioritized their form rather than their content or presumed etiology [30]. This approach aligned with a broader mid-century emphasis on descriptive phenomenology and operational clinical observation.

The primary significance of Schneider’s first-rank symptoms lay in their proposed diagnostic specificity. Schneider argued that, when present, these symptoms could distinguish schizophrenia from other psychotic conditions, particularly affective psychoses [31]. As a result, first-rank symptoms gained substantial influence in clinical practice and were later incorporated into international diagnostic systems, including early editions of the DSM and ICD. Their apparent ease of identification made them attractive tools for improving inter-rater reliability in psychiatric diagnosis [32].

However, subsequent empirical research revealed important limitations of the first-rank symptom approach. Studies demonstrated that these symptoms were neither universally present in schizophrenia nor exclusive to it, as they could also be observed in mood disorders with psychotic features and other psychiatric conditions [33]. Moreover, the focus on a limited set of striking psychotic phenomena risked marginalizing negative symptoms and cognitive impairments, which increasingly came to be recognized as central to long-term functional outcome.

**Figure 2:**
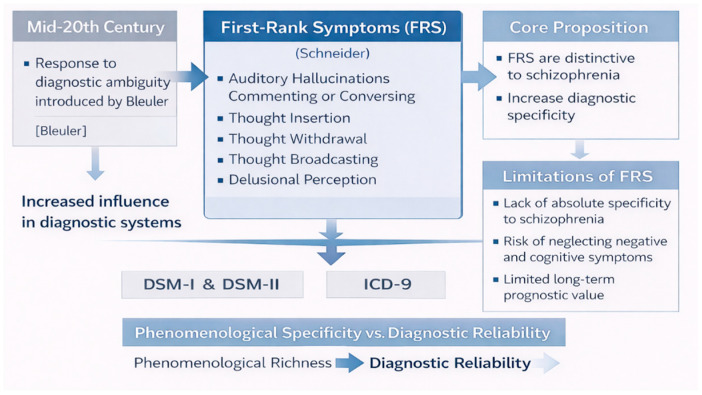
**Schneider and first-rank symptoms in the evolution of schizophrenia diagnosis**. The figure summarizes Kurt Schneider’s introduction of first-rank symptoms as phenomenological markers aimed at improving diagnostic specificity and reliability, and illustrates their historical influence and subsequent limitations within psychiatric classification.

From a historical perspective, Schneider’s contribution represents a critical intermediate stage between Bleuler’s broad, theory-laden concept of schizophrenia and the later operationalized diagnostic criteria of the DSM-III era. His work exemplifies the enduring tension between phenomenological richness and diagnostic reliability, a tension that continues to shape contemporary debates in brain sciences [34]. While first-rank symptoms ultimately failed to provide definitive diagnostic markers, their influence underscores the persistent search for clinically observable features capable of anchoring schizophrenia as a distinct diagnostic entity. Subsequent analyses demonstrated that first-rank symptoms lacked both sensitivity and specificity, limiting their diagnostic utility and anticipating later critiques of categorical diagnosis [35]. Their apparent ease of identification made them attractive tools for improving inter-rater reliability in psychiatric diagnosis (Figure 2).

## 6. Early Classification Systems

The progressive consolidation of schizophrenia as a diagnostic entity during the twentieth century occurred in parallel with the development of formal psychiatric classification systems. Early nosographic efforts aimed to standardize diagnostic language and improve communication across clinicians, institutions, and national contexts [36]. In this phase, classification systems functioned less as reflections of underlying disease mechanisms and more as pragmatic tools for organizing clinical observations and administrative practices [10].

The first editions of the *Diagnostic and Statistical Manual of Mental Disorders* (DSM-I, 1952) and DSM-II (1968) reflected this descriptive and largely theory-neutral approach. Schizophrenia was broadly defined, encompassing a wide range of psychotic presentations and subtypes, including paranoid, catatonic, hebephrenic, and undifferentiated forms. Diagnostic criteria relied heavily on clinical judgment and narrative description rather than explicit operational rules, resulting in substantial variability in diagnostic application across clinicians and settings [37,38]. Similar principles guided early versions of the *International Classification of Diseases* (ICD), which likewise emphasized descriptive categories over etiological assumptions.

These early classification systems were strongly influenced by psychoanalytic and psychodynamic frameworks, particularly in North America, where schizophrenia was often conceptualized as a reaction to environmental stressors or intrapsychic conflict. As a consequence, diagnostic boundaries remained fluid, and the distinction between schizophrenia and affective or personality disorders was frequently blurred. While this flexibility allowed for clinical nuance, it also compromised diagnostic reliability and posed challenges for epidemiological research and comparative studies.

By the mid-twentieth century, growing dissatisfaction with diagnostic inconsistency became increasingly evident. Studies comparing diagnostic practices across countries revealed striking discrepancies in the prevalence and conceptualization of schizophrenia, underscoring the limitations of loosely defined diagnostic categories [39]. These concerns were amplified by the expanding role of clinical trials and biological research, which required more homogeneous and reproducible patient populations.

From a historical perspective, DSM-I, DSM-II, and early ICD editions represent a transitional phase in the evolution of psychiatric nosography. They formalized schizophrenia as an official diagnostic category while simultaneously exposing the inherent tensions between descriptive richness, theoretical pluralism, and the need for standardization. The shortcomings of these early systems ultimately set the stage for the operational revolution of the DSM-III era, which sought to prioritize diagnostic reliability through explicit criteria and symptom checklists [40].

## 7. DSM-III and the Operational Revolution

The publication of the third edition of the Diagnostic and Statistical Manual of Mental Disorders (DSM-III) in 1980 marked a profound rupture in the history of psychiatric classification and fundamentally reshaped the diagnostic framework of schizophrenia. This transformation did not emerge from new discoveries regarding etiology or pathophysiology, but rather from growing institutional, scientific, and political concerns about the lack of diagnostic reliability that characterized earlier classification systems. In this context, DSM-III represented a strategic effort to reposition psychiatry as a scientifically credible medical discipline through the adoption of explicit, operationalized diagnostic criteria [41].

For schizophrenia, DSM-III replaced loosely defined, theory-laden descriptions with symptom checklists, duration thresholds, and exclusion rules designed to maximize inter-rater reliability. This shift significantly reduced the influence of psychodynamic interpretation and phenomenological depth, prioritizing observable and reportable symptoms over inferred psychological structures [42]. However, the operational emphasis of DSM-III also contributed to the relative marginalization of negative and cognitive symptoms, which were less easily operationalized compared to positive psychotic features [43,44]. As a result, diagnostic criteria prioritized observable psychotic symptoms, potentially overlooking dimensions more closely related to functional outcome and long-term disability [45]. This imbalance influenced both clinical research and therapeutic development, reinforcing a focus on acute symptom reduction rather than broader functional recovery [46]. While Schneiderian first-rank symptoms continued to inform diagnostic thinking, they were no longer regarded as pathognomonic, reflecting an explicit move toward descriptive neutrality and operational clarity [47].

The operational revolution introduced by DSM-III had far-reaching consequences for research and clinical practice. Standardized diagnostic criteria enabled large-scale epidemiological studies, facilitated multicenter clinical trials, and supported the rapid expansion of psychopharmacological research [38,48]. At the same time, critics argued that DSM-III transformed provisional clinical constructs into reified disease entities, thereby obscuring conceptual uncertainties and biological heterogeneity within diagnostic categories such as schizophrenia [49].

**Figure 3:**
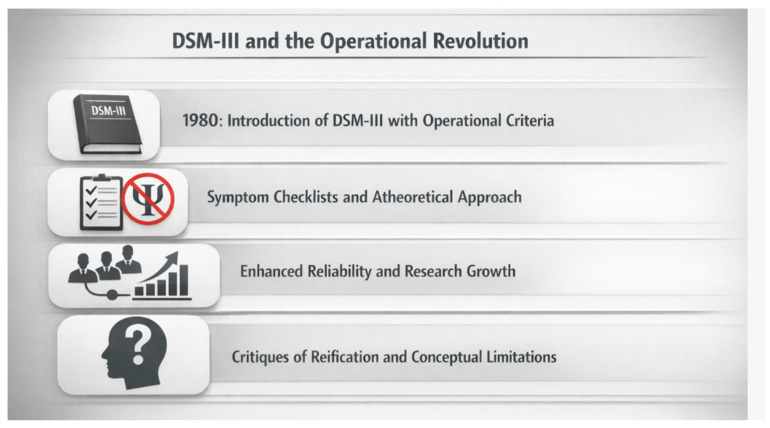
**DSM-III and the operational revolution in psychiatric diagnosis.** Schematic overview of the shift toward operationalized, symptom-based diagnostic criteria introduced with DSM-III, highlighting gains in reliability alongside emerging conceptual limitations.

From a historical standpoint, DSM-III can be understood as an institutional solution to a crisis of legitimacy rather than a resolution of long-standing conceptual debates about the nature of mental illness. Its deliberately atheoretical stance allowed psychiatry to sidestep unresolved questions of causation, but at the cost of narrowing the clinical gaze and marginalizing aspects of psychopathology that resisted operationalization [50]. Subsequent critiques have highlighted how this approach contributed to the erosion of phenomenological traditions and reinforced categorical models that remain difficult to reconcile with emerging findings in neuroscience and genetics (Figure 3).

Despite multiple revisions, the operational framework introduced by DSM-III continues to shape contemporary diagnostic practice. Its legacy persists as both a methodological achievement and a conceptual constraint, framing ongoing debates in brain sciences about validity, dimensionality, and the future of schizophrenia as a diagnostic category [51].

## 8. Modern Diagnostic Systems (DSM-IV to DSM-5, ICD-10 to ICD-11)

The period spanning the late twentieth and early twenty-first centuries was characterized by incremental yet conceptually significant revisions of diagnostic systems for schizophrenia. Following the operational revolution initiated by DSM-III, subsequent editions sought to refine diagnostic criteria while preserving the core categorical framework. DSM-IV and DSM-IV-TR largely maintained the symptom-based structure introduced in 1980, emphasizing reliability and continuity over radical conceptual change. Schizophrenia remained defined by a combination of positive, negative, and disorganized symptoms, alongside duration and functional impairment criteria, reinforcing its status as a discrete diagnostic entity [52].

During this phase, growing awareness of the limitations of categorical diagnosis began to influence revision processes. Accumulating evidence highlighted substantial overlap between schizophrenia and other psychotic and affective disorders, as well as marked heterogeneity within the diagnosis itself. Longitudinal studies demonstrated variable illness trajectories, challenging assumptions of uniform progression and outcome [53]. Despite these findings, DSM-IV preserved traditional subtypes (paranoid, catatonic, disorganized, undifferentiated, residual), reflecting a cautious approach to nosographic change.

A more explicit reassessment emerged with the development of DSM-5. One of the most notable revisions was the elimination of schizophrenia subtypes, which were found to have limited diagnostic stability, poor prognostic value, and minimal relevance for treatment selection [54]. In their place, DSM-5 introduced dimensional symptom severity assessments intended to capture within-diagnosis variability. This shift acknowledged the inadequacy of rigid categorical boundaries while stopping short of abandoning the diagnostic construct itself [25].

Parallel developments occurred within the ICD framework. ICD-10 largely mirrored DSM-IV in its categorical structure, but the revision process leading to ICD-11 placed greater emphasis on clinical utility, global applicability, and dimensional description. ICD-11 incorporated a more flexible characterization of schizophrenia, focusing on symptom domains and course specifiers rather than fixed subtypes [55]. These changes reflected an effort to reconcile diagnostic standardization with clinical heterogeneity across cultural and healthcare contexts [56].

Concurrently, alternative conceptual models gained prominence. Dimensional and spectrum-based approaches challenged the categorical separation of psychotic disorders, proposing continua that span schizophrenia, schizoaffective disorder, bipolar disorder, and related conditions [57]. Staging models further emphasized illness progression, early intervention, and risk states, reframing schizophrenia as a dynamic process rather than a static diagnosis [58]. These frameworks resonated with findings from genetics and neuroscience, which increasingly pointed to shared vulnerability factors across traditional diagnostic categories.

Despite these advances, modern diagnostic systems continue to face fundamental challenges. Dimensional measures remain secondary to categorical diagnoses in clinical and research practice, and no consensus has emerged regarding the optimal integration of biological markers into diagnostic criteria [59]. Initiatives such as the Research Domain Criteria (RDoC) have sought to move beyond symptom-based classification altogether, but their impact on routine diagnosis remains limited [60].

From a historical perspective, DSM-5 and ICD-11 represent evolutionary rather than revolutionary steps in the classification of schizophrenia. They reflect growing recognition of heterogeneity, dimensionality, and biological complexity, while remaining anchored to the categorical structures established in the twentieth century. This tension underscores the enduring influence of historical diagnostic constructs on contemporary brain sciences and highlights the challenges inherent in reconciling clinical practice with emerging scientific evidence.

## 9. Diagnostic Instruments and Clinical Scales

The operationalization of psychiatric diagnosis introduced with DSM-III not only reshaped nosographic systems but also stimulated the development and widespread adoption of standardized diagnostic instruments and clinical rating scales. These tools were designed to translate abstract diagnostic criteria into reproducible assessments, thereby improving diagnostic reliability, facilitating research comparability, and supporting large-scale clinical trials. In the context of schizophrenia, diagnostic instruments became essential mediators between nosographic definitions and clinical practice [61].

**Figure 4:**
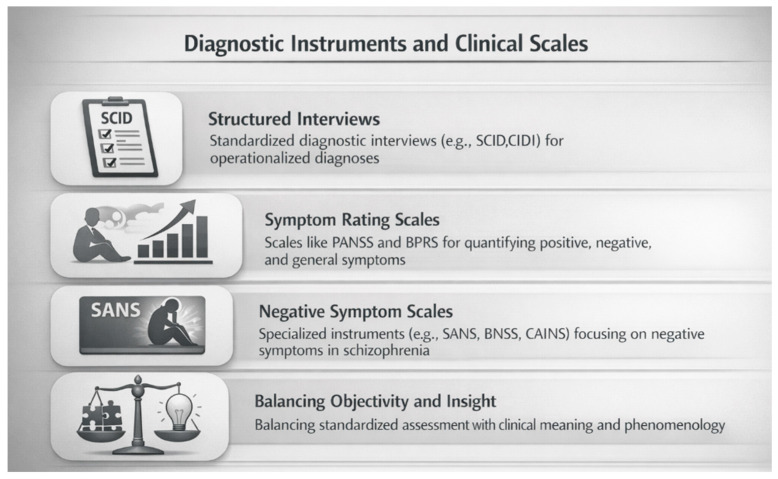
**Diagnostic instruments and clinical scales in schizophrenia.** Overview of structured interviews and symptom rating scales developed to operationalize diagnosis and quantify symptom domains.

Structured and semi-structured interviews represented a key methodological innovation. Instruments such as the Structured Clinical Interview for DSM (SCID) and the Composite International Diagnostic Interview (CIDI) aimed to reduce subjective variability by standardizing symptom assessment and diagnostic decision-making [62]. While initially developed for research purposes, these tools increasingly influenced clinical settings, reinforcing the primacy of operational criteria over traditional clinical judgment (Figure 4).

Parallel to diagnostic interviews, symptom rating scales emerged as central tools for characterizing illness severity and treatment response. The Brief Psychiatric Rating Scale (BPRS) and the Positive and Negative Syndrome Scale (PANSS) became widely used instruments for quantifying psychotic symptoms across multiple domains [63]. These scales reflected the growing recognition that schizophrenia could not be adequately captured by a single diagnostic label, but required multidimensional assessment of symptom profiles.

The introduction of negative symptom scales, such as the Scale for the Assessment of Negative Symptoms (SANS), marked an important conceptual shift. Negative symptoms, long overshadowed by more conspicuous psychotic phenomena, were increasingly recognized as core features of schizophrenia with major implications for functional outcome [64]. More recently, revised instruments such as the Brief Negative Symptom Scale (BNSS) and the Clinical Assessment Interview for Negative Symptoms (CAINS) have sought to address limitations of earlier scales, including poor construct validity and overlap with depression or medication effects [65].

Despite their methodological advantages, diagnostic instruments and rating scales also introduced new challenges. The emphasis on quantification risked fragmenting clinical phenomena into discrete items, potentially obscuring the experiential coherence emphasized in phenomenological traditions [66]. Moreover, scale-based assessments often reflected the theoretical assumptions embedded in prevailing diagnostic frameworks, reinforcing categorical models rather than challenging them. In addition, modern rating scales present important conceptual and methodological limitations. Several symptom domains assessed by instruments such as the PANSS and BPRS overlap with other neuropsychiatric conditions, including mood disorders, neurodevelopmental disorders, and neurodegenerative diseases [67]. Negative symptoms, in particular, may overlap with depressive symptoms, medication effects, or cognitive impairment, reducing diagnostic specificity [68]. Furthermore, cross-sectional rating scales may inadequately capture longitudinal variability, environmental influences, and functional outcomes. These limitations highlight the need for multimodal assessment strategies integrating clinical, biological, and digital measures. From a historical perspective, diagnostic instruments and clinical scales can be seen as both products and drivers of the operational paradigm. They enhanced reliability and facilitated research progress, yet also contributed to the reification of diagnostic constructs.

Their continued evolution reflects ongoing efforts to balance standardization with clinical meaning, a tension that remains central to contemporary brain sciences and the study of schizophrenia [69].

## 10. Biomarkers and the Search for Biological Validity

From the late twentieth century onward, increasing dissatisfaction with purely symptom-based diagnostic systems prompted an intensified search for biological markers capable of grounding schizophrenia in objectively measurable pathophysiological processes. This effort was motivated by the hope that biomarkers could resolve longstanding questions regarding diagnostic validity, reduce heterogeneity, and align psychiatric classification more closely with other areas of medicine. In this context, schizophrenia became a central target for biomarker research, reflecting both its clinical burden and its conceptual instability [70].

Early biomarker investigations focused primarily on neurochemical hypotheses, particularly dopaminergic dysfunction. While these models provided important insights into antipsychotic drug mechanisms, they failed to yield diagnostic markers with sufficient specificity or sensitivity. As a result, attention shifted toward neuroimaging techniques, including structural and functional magnetic resonance imaging (MRI and fMRI), which revealed consistent group-level differences in brain volume, connectivity, and activation patterns in individuals diagnosed with schizophrenia [71]. However, these findings exhibited substantial overlap with other psychiatric conditions and significant variability across patients.

Genetic research further complicated the biomarker landscape. Genome-wide association studies (GWAS) identified numerous risk loci associated with schizophrenia, implicating synaptic, neurodevelopmental, and immune-related pathways [72]. Yet, the polygenic architecture of these findings undermined the notion of a single or even a limited set of diagnostic biomarkers. Instead, genetic risk appeared to be widely shared across traditional diagnostic boundaries, reinforcing dimensional and spectrum-based models of psychopathology [73].

Parallel efforts explored peripheral biomarkers, including inflammatory markers, oxidative stress indicators, and neuroendocrine measures. Although alterations in immune and inflammatory pathways were repeatedly observed in subsets of patients, these markers lacked consistency and diagnostic exclusivity [74]. From a mechanistic perspective, neuroinflammation has increasingly been linked to neurodevelopmental models of schizophrenia [75,76]. Microglial activation, triggered by early-life immune challenges or later environmental stressors, has been proposed as a key mechanism underlying synaptic dysfunction and gray matter volume reduction [77,78]. Within the “two-hit” neurodevelopmental model, early vulnerability interacts with later inflammatory or environmental insults, leading to aberrant synaptic pruning and circuit-level alterations [79,80,81]. Reactive microglia, through complement-mediated synaptic phagocytosis, may contribute to progressive cortical thinning and network dysconnectivity [82,83]. These mechanisms support the emergence of biologically meaningful inflammatory subtypes, suggesting that stratification based on immune-related pathways may represent a promising direction for future research [84].

**Figure 5:**
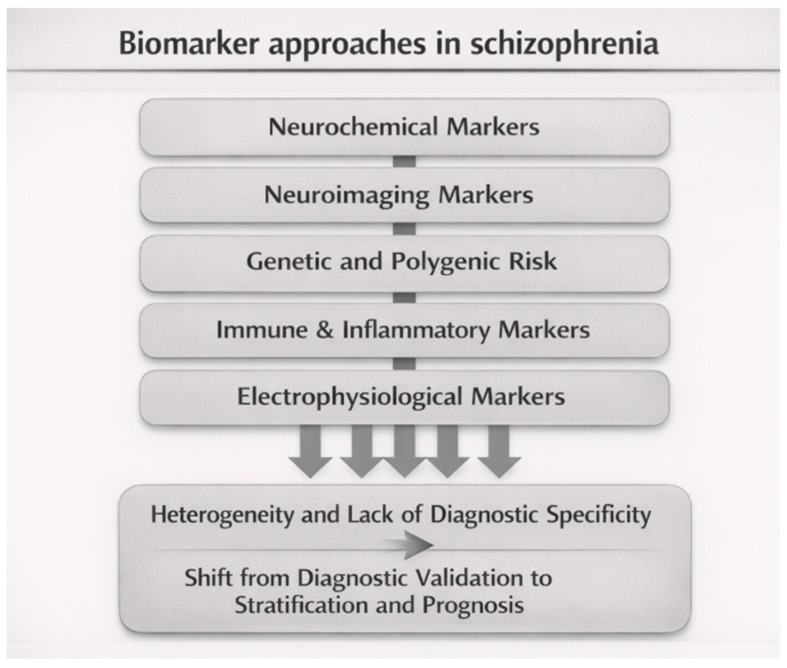
**Biomarker approaches in schizophrenia.** Conceptual overview of major biomarker strategies and their convergence toward heterogeneity and limited diagnostic specificity, highlighting the shift from diagnostic validation to stratification- and prognosis-oriented models. The figure also illustrates potential stratification approaches based on multimodal biomarkers, including inflammatory, neurodevelopmental, and connectivity-based subtypes.

Similarly, electrophysiological measures such as mismatch negativity and P300 abnormalities demonstrated promise as indicators of cognitive dysfunction, but fell short of serving as definitive diagnostic tools (Figure 5) [85]. Emerging biomarker research has also focused on inflammatory pathways, with cytokine alterations including interleukin-6, tumor necrosis factor-α, and C-reactive protein repeatedly associated with symptom severity and disease progression [74,86]. In parallel, electrophysiological measures such as mismatch negativity and P300 abnormalities have been proposed as neurophysiological markers of cognitive dysfunction [87,88]. Resting-state functional connectivity studies further identified alterations in default mode, salience, and executive networks [89]. However, these findings remain non-specific and frequently overlap with other psychiatric and neurological conditions, limiting their diagnostic utility.

More recently, multimodal and machine learning approaches have sought to integrate neuroimaging, genetic, cognitive, and clinical data to identify biologically meaningful subtypes of schizophrenia. These approaches aim to move beyond single-marker strategies by capturing complex patterns associated with illness mechanisms and outcome trajectories [90]. While promising, such models remain largely confined to research settings and have yet to translate into routine diagnostic practice.

From a historical perspective, the persistent failure to identify definitive biomarkers for schizophrenia reflects not only technical limitations but also the inherited ambiguity of the diagnostic construct itself. Biomarker research has repeatedly exposed the mismatch between categorical diagnoses and underlying biological continua [91]. Rather than resolving the diagnostic problem, biomarkers have often mirrored the heterogeneity embedded within existing classification systems.

In contemporary brain sciences, biomarkers are increasingly viewed as tools for stratification, prognosis, and treatment response rather than as diagnostic validators. This reconceptualization marks a significant shift away from the original goal of biological legitimation toward a more pragmatic integration of biological data within dimensional and transdiagnostic frameworks [92]. As such, the history of biomarker research in schizophrenia underscores the need to reconsider the relationship between diagnosis, biology, and clinical utility [93].

## 11. Omics, Systems Biology, and Endophenotypes

From The limitations encountered in single-marker biomarker research prompted a progressive shift toward integrative biological frameworks aimed at capturing the complexity of schizophrenia. Rather than seeking discrete diagnostic indicators, contemporary research increasingly adopted *omics* technologies and systems biology approaches to explore multilevel interactions among genes, transcripts, proteins, metabolites, and environmental factors. This transition reflected a broader reconceptualization of schizophrenia as a disorder emerging from dysregulated biological networks rather than isolated molecular defects [94].

Genomic and transcriptomic studies expanded beyond risk locus identification to examine gene expression profiles, epigenetic modifications, and regulatory mechanisms across brain and peripheral tissues. Transcriptomic analyses revealed widespread dysregulation of synaptic, immune, and neurodevelopmental pathways, but also underscored substantial inter-individual variability [95]. Epigenetic findings, including DNA methylation and histone modification patterns, further highlighted the dynamic interplay between genetic vulnerability and environmental exposures, challenging static disease models [96].

Proteomics and metabolomics provided complementary perspectives by interrogating downstream molecular processes more closely related to cellular function. Alterations in energy metabolism, oxidative stress pathways, lipid signaling, and neurotransmitter-related metabolites were repeatedly reported in schizophrenia cohorts [97]. However, as with earlier biomarker efforts, these findings rarely demonstrated diagnostic specificity and were strongly influenced by medication status, illness stage, and comorbidities.

Systems biology approaches sought to integrate these diverse datasets into coherent models of disease mechanisms. Network-based analyses identified convergent pathways involving synaptic plasticity, immune signaling, and neurodevelopmental regulation, offering a more holistic view of pathophysiology [98]. Importantly, these approaches shifted the analytical focus from categorical diagnosis to biological stratification, enabling the identification of subgroups characterized by shared molecular profiles rather than shared diagnostic labels.

Within this context, the concept of *endophenotypes* gained renewed relevance. Endophenotypes heritable, quantifiable traits positioned between genotype and clinical phenotype, were proposed as more biologically tractable targets than heterogeneous diagnostic categories [99]. Cognitive deficits, neurophysiological markers, and specific neuroimaging patterns were extensively investigated as potential endophenotypes for schizophrenia. While some demonstrated familial aggregation and state independence, their relationship to clinical diagnosis remained complex and non-exclusive [100].

Despite their theoretical appeal, omics-based stratification and endophenotype approaches faced significant challenges. Reproducibility across cohorts remained limited, and the translation of molecular subtypes into clinically actionable frameworks proved difficult [72]. Moreover, the persistence of diagnostic categories in clinical practice constrained the integration of systems-level findings into routine care.

**Figure 6:**
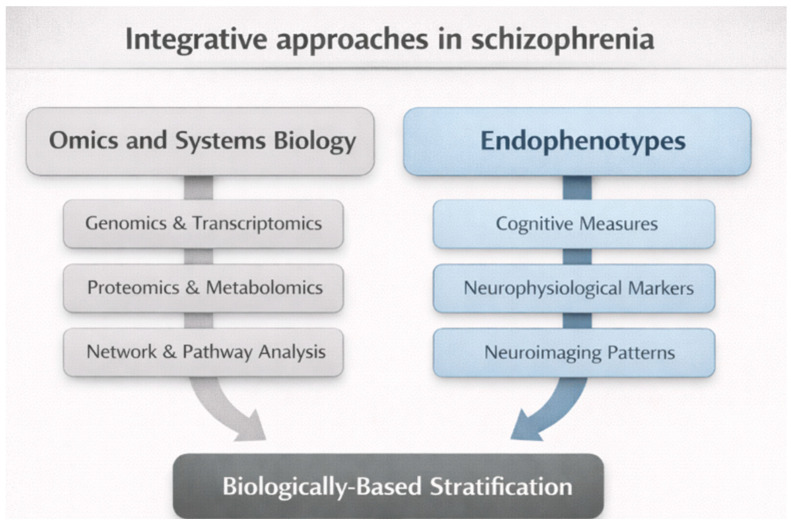
**Integrative omics, systems biology, and endophenotype frameworks in schizophrenia.** Schematic representation of how multi-omics and systems biology approaches (genomics, transcriptomics, proteomics, metabolomics, and network analyses) converge with endophenotypic measures (cognitive, neurophysiological, and neuroimaging markers) to support biologically based stratification beyond categorical diagnosis. These frameworks support biologically informed stratification models, moving from categorical diagnosis toward multimodal subtype identification.

From a historical perspective, the rise of omics and systems biology represents not a break from earlier diagnostic paradigms, but an adaptive response to their limitations. A further limitation of omics-based approaches concerns a potential circularity problem. Most large-scale genomic, transcriptomic, and multi-omics studies rely on DSM- or ICD-based diagnoses for sample selection [72,101]. However, these diagnostic categories are historically derived and biologically heterogeneous constructs [102]. As a result, biological investigations may attempt to identify molecular markers within populations that are already heterogeneous by definition. This circularity may partially explain the inconsistent and non-specific findings observed across omics studies [103,104]. Rather than identifying unified biological signatures, these approaches frequently reveal overlapping and transdiagnostic patterns, reflecting the limitations of historically derived diagnostic scaffolds [60]. These approaches exposed the inadequacy of categorical models while simultaneously depending on them for sample selection and interpretation [105]. As such, they occupy an intermediate epistemic position, advancing biological insight without fully resolving the conceptual tensions surrounding schizophrenia (Figure 6).

In contemporary brain sciences, omics and systems biology increasingly serve as tools for hypothesis generation, risk stratification, and treatment personalization rather than diagnostic validation. Their future impact will depend on the development of integrative frameworks capable of bridging biological complexity with clinical meaning, a challenge that remains deeply shaped by the historical evolution of schizophrenia as a diagnostic construct [93].

## 12. Digital Psychiatry and Computational Approaches in the Digital Era

A central Over the past decade, schizophrenia research has undergone a marked shift driven by the consolidation of the digital era in medicine. This transformation extends beyond artificial intelligence alone and includes digital phenotyping, telepsychiatry, real-world data infrastructures, and computational psychiatry. Together, these developments have expanded the measurable phenotype of schizophrenia from episodic clinic-based assessments to longitudinal, ecologically valid data streams collected in daily life [106,107].

Digital phenotyping continuous, real-world data collection via smartphones, wearables, and ecological momentary assessment has emerged as a promising approach for capturing symptom dynamics outside clinical settings. Longitudinal patterns of activity, social engagement, speech, and sleep have been associated with symptom worsening and relapse risk, offering a non-invasive strategy for monitoring illness trajectories [106,107,108,109]. Landmark work using passively collected smartphone data demonstrated that behavioral changes can precede relapse and may be detectable through deviations (“anomalies”) in individualized baselines [109]. Subsequent prospective multi-site work has reinforced feasibility and extended this approach to open-source platforms (e.g., mindLAMP), supporting the use of digital signals for relapse-relevant monitoring in real-world settings [110]. Methodological analyses of large digital phenotyping cohorts further emphasize the importance of standardization, missingness handling, and clinically interpretable feature engineering in order to improve reproducibility and utility [111].

In parallel, computational psychiatry has matured as a framework combining machine learning with generative and predictive models to identify latent structures in high-dimensional clinical and biological data. Rather than serving as mere diagnostic classifiers, contemporary models increasingly focus on prediction (e.g., relapse probability, functional decline) and stratification, aligning with the broader move from categorical labeling toward clinically meaningful risk modeling [108,112]. Reviews of machine learning in clinical psychiatry highlight both the potential multimodal integration and individualized prediction and persistent limitations, including overfitting, site effects, and limited transportability across populations [112].

The digital era has also reshaped care delivery through the rapid expansion of telepsychiatry and hybrid models, especially after 2020. Telepsychiatry has improved access and continuity for follow-up care, yet evidence also notes variable acceptability and perceived effectiveness depending on clinical purpose, patient needs, and context [113]. These service-level shifts matter historically because they alter the “ecology” of clinical observation and generate new forms of structured data that increasingly feed research pipelines.

**Figure 7:**
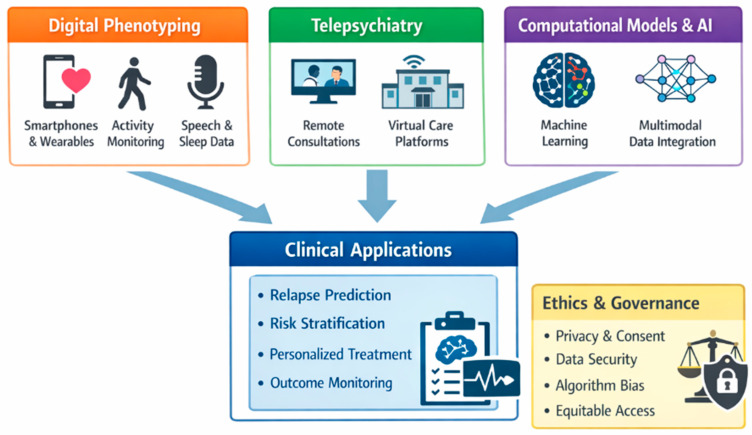
**Digital psychiatry and computational approaches in schizophrenia.** Schematic overview of digital phenotyping, telepsychiatry, and AI-driven computational models converging toward predictive, stratified, and ethically governed clinical applications.

Despite rapid progress, ethical, privacy, and governance questions remain central. Consensus efforts and practical instruments propose safeguards for digital phenotyping and digital mental health research covering consent, equity, privacy, data partnerships, and duty-to-warn procedures reflecting the distinctive risks of continuous monitoring and algorithmic inference in severe mental illness [114,115]. Recent viewpoints also stress that digital phenotyping should augment, not displace, clinical expertise and therapeutic relationships [116]. Despite growing enthusiasm, digital psychiatry faces important methodological and translational limitations. Digital phenotyping approaches often suffer from signal-to-noise variability, missing data, and heterogeneity across devices and platforms [117,118]. In addition, behavioral signals derived from smartphones or wearable devices may be influenced by contextual and environmental factors unrelated to illness progression [119]. These challenges limit reproducibility across cohorts and currently constrain the use of digital tools for individualized treatment decisions [120]. Furthermore, the lack of standardization across digital infrastructures complicates integration into routine clinical practice [121]. As a result, digital psychiatry remains a promising but still evolving field requiring further validation before widespread clinical implementation [122]. From a historical lens, digital and computational approaches represent the latest phase in psychiatry’s long pursuit of operationalization and objectification now reframed around longitudinal measurement, probabilistic prediction, and responsible implementation rather than definitive diagnostic proofs (Figure 7).

## 13. Discussion: Historical Trajectories, Validity, and the Future of Diagnosis

The historical development of schizophrenia reveals a persistent dialectic between descriptive stabilization and conceptual instability. Despite the rapid expansion of systems neuroscience and circuit-level knowledge, the translation of mechanistic insights into effective therapies for schizophrenia has remained remarkably slow. This translational gap, often referred to as the “valley of death”, reflects not only biological complexity but also limitations in current diagnostic frameworks [27,123,124]. Contemporary drug development frequently relies on heterogeneous patient populations defined by categorical DSM or ICD diagnoses that lack clear biological boundaries [104,125]. As a result, proof-of-concept trials may fail to detect treatment effects in biologically responsive subgroups. A recent example is represented by TAAR1 agonists, which showed promising mechanistic rationale but failed in Phase III clinical trials, partly due to high placebo responses and unselected patient populations [126,127,128]. These challenges suggest that historically derived diagnostic categories may actively hinder translational progress, reinforcing the need for biologically informed stratification approaches [129,130]. While diagnostic systems have progressively improved reliability, questions regarding validity and biological coherence remain unresolved. Recent scholarship emphasizes that psychiatric categories, including schizophrenia, function as pragmatic constructs shaped by evolving scientific, institutional, and technological contexts rather than as fixed natural kinds [131,132].

Contemporary critiques highlight that operational diagnostic systems, although essential for standardization, may reify heterogeneous syndromes whose biological substrates are distributed across overlapping neurodevelopmental and network-based processes [133,134]. Advances in psychiatric genomics have demonstrated substantial polygenic overlap between schizophrenia and other psychiatric conditions, challenging strict categorical boundaries and reinforcing dimensional liability models [135]. Similarly, neuroimaging meta-analyses reveal distributed patterns of dysconnectivity rather than disease-specific structural signatures [136].

The emergence of systems neuroscience and network medicine frameworks further reframes schizophrenia as a perturbation of interacting biological systems rather than a discrete pathological entity [137]. Within this perspective, heterogeneity is not a failure of classification but an intrinsic feature of complex brain disorders. Such findings align with proposals advocating for pluralistic and layered explanatory models in psychiatry, integrating biological, psychological, and social determinants without reducing one level to another [91].

Digital psychiatry and AI-driven computational approaches intensify this epistemic shift. Rather than seeking categorical confirmation, recent work prioritizes predictive performance, stratification, and longitudinal risk modeling [138]. These models often demonstrate that symptom dimensions and outcome trajectories provide greater explanatory and predictive utility than traditional diagnoses alone. However, critical analyses caution against technological solutionism, emphasizing the need for transparency, fairness, and clinical interpretability in algorithmic systems [139].

Importantly, contemporary discussions increasingly distinguish between clinical utility and ontological validity. A diagnostic category may remain clinically useful even if its biological boundaries are porous or context-dependent [140]. In this sense, schizophrenia persists not because it represents a fully validated biological disease entity, but because it provides a functional scaffold for organizing care, research, and communication.

From a historical perspective, schizophrenia can thus be understood as a dynamic epistemic construct that evolves in response to methodological innovation. Each technological era descriptive psychopathology, operational nosology, biomarker research, omics integration, and digital modeling has reshaped but not replaced the concept. Rather than signaling failure, this adaptability may reflect the inherent complexity of severe mental disorders.

Future progress in brain sciences will likely depend on integrating categorical, dimensional, biological, and digital models into flexible frameworks capable of accommodating heterogeneity without abandoning clinical clarity. The historical trajectory of schizophrenia suggests that conceptual refinement, rather than categorical elimination, remains the most plausible path forward [141].

## 14. Conclusions and Future Directions

The historical evolution of schizophrenia demonstrates that psychiatric concepts are neither static nor purely descriptive, but dynamic constructs shaped by shifting scientific paradigms, methodological tools, and institutional needs. From pre-nosographic interpretations of madness to Kraepelin’s longitudinal disease model, from Bleuler’s reconceptualization of core psychic disturbances to Schneider’s phenomenological criteria, and from DSM operationalization to contemporary biomarker and digital frameworks, each era has redefined the boundaries and meaning of the disorder. Yet no single transformation has fully resolved the fundamental tension between clinical coherence and biological heterogeneity.

Operational diagnostic systems, particularly since DSM-III, achieved substantial gains in reliability and research standardization. However, advances in genetics, neuroimaging, and systems neuroscience have repeatedly shown that schizophrenia lacks a singular, disease-specific biological signature. Instead, it reflects distributed risk architectures, overlapping neurodevelopmental pathways, and network-level perturbations that extend across traditional diagnostic boundaries. Omics technologies and systems biology further reinforced this complexity, revealing molecular convergence without diagnostic exclusivity.

The digital and computational era has introduced a further conceptual shift. Rather than seeking categorical validation, contemporary approaches increasingly prioritize longitudinal monitoring, probabilistic prediction, and clinically meaningful stratification. Digital phenotyping, telepsychiatry, and AI-driven modeling extend psychiatric assessment beyond episodic encounters, embedding diagnosis within continuous and context-sensitive data streams. These tools do not abolish the diagnostic construct but transform its operational environment, reframing schizophrenia as a dynamic risk profile rather than a fixed entity.

Importantly, this historical trajectory suggests that the future of schizophrenia research lies not in replacing existing categories outright, but in integrating multiple explanatory layers clinical, biological, dimensional, and computational within flexible frameworks. The distinction between clinical utility and ontological validity becomes central: a diagnosis may remain useful for communication, treatment planning, and research organization even if its biological boundaries are porous.

Future directions should therefore emphasize reproducible multimodal integration, transparent and ethically governed AI applications, and translational models that bridge biological stratification with individualized care. At the same time, continued conceptual reflection remains essential. Technological sophistication alone cannot substitute for careful epistemological analysis.

Schizophrenia endures as a historically evolving construct—one that reflects the complexity of severe mental illness and the adaptive interplay between concept, method, and evidence. Its future will likely be defined not by categorical dissolution, but by progressive refinement toward models that respect heterogeneity while preserving clinical clarity.

## Data Availability

No new data were created or analyzed in this study. Data sharing is not applicable to this article.

## Abbreviations

The following abbreviations are used in this manuscript:

AI	Artificial Intelligence
APC	Article Processing Charge
BNSS	Brief Negative Symptom Scale
BPRS	Brief Psychiatric Rating Scale
CAINS	Clinical Assessment Interview for Negative Symptoms
CIDI	Composite International Diagnostic Interview
COVID-19	Coronavirus Disease 2019
DNA	Deoxyribonucleic Acid
DSM	Diagnostic and Statistical Manual of Mental Disorders
DSM-I	Diagnostic and Statistical Manual of Mental Disorders, First Edition
DSM-II	Diagnostic and Statistical Manual of Mental Disorders, Second Edition
DSM-III	Diagnostic and Statistical Manual of Mental Disorders, Third Edition
DSM-IV	Diagnostic and Statistical Manual of Mental Disorders, Fourth Edition
DSM-5	Diagnostic and Statistical Manual of Mental Disorders, Fifth Edition
FRS	First-Rank Symptoms
GM	Gray Matter
GWAS	Genome-Wide Association Studies
ICD	International Classification of Diseases
ICD-10	International Classification of Diseases, 10th Revision
ICD-11	International Classification of Diseases, 11th Revision
KS	Kurt Schneider
MRI	Magnetic Resonance Imaging
PANSS	Positive and Negative Syndrome Scale
SANS	Scale for the Assessment of Negative Symptoms
SCID	Structured Clinical Interview for DSMs
